# Trial of a Trivial Quantitative Heat-Pain Stimulus for Detecting Severe Loss of Nociception

**DOI:** 10.1177/19322968221144328

**Published:** 2022-12-22

**Authors:** Ernst-Adolf Chantelau, Oliver Schröer

**Affiliations:** 1 Heinrich Heine University Düsseldorf, Düsseldorf, Germany; 2 Outpatient Diabetic Foot Clinic, St. Martinus-Krankenhaus Düsseldorf, Düsseldorf, Germany

**Keywords:** diabetic neuropathy, pain insensitivity, diagnostic test, quantitative sensory testing

## Abstract

**Background:**

Loss of nociception (LON) at the feet of persons with diabetes mellitus develops gradually over years and remains asymptomatic until the first painless diabetic foot ulceration (DFU). Severe LON with pain insensitivity can be diagnosed with a mechanical (pinprick) pain stimulus of 512-mN force. A comparable “suprathreshold” heat-pain stimulus may have the same potential.

**Objective:**

A six-second, 51°C heat-pain stimulus delivered on a 38.5-mm² spot by a commercial medical device (*bite away®*, to treat insect bites) was explored in a prospective cross-sectional diagnostic accuracy study to detect DFU-related LON.

**Methods:**

Seventy-two participants were studied: 12 with and 30 without diabetic neuropathy according to the conventional criteria, and 30 patients with a history of painless DFU (indicative of end-stage LON, reference standard). The feet were stimulated at the plantar and dorsal sides. A palmar surface was stimulated for control purposes. Participants scored stimulated pain intensity 0 to 10 on a numerical rating scale.

**Results:**

At hands, pain intensity was rated six on average by all participants. Persons without neuropathy scored 7 (0-10), median (range), at the plantar side and 8.5 (2-10) at the dorsal side of the foot, while those with DFU scored 0 (0-8) and 0 (0-10), respectively. A pain response of 0 at the foot dorsum detected DFU-related LON with a sensitivity of 65% (specificity, 100%; positive and negative predictive values, 100% and 96%, respectively).

**Conclusions:**

Due to its high specificity, the test seems advantageous for diagnostic purposes, complementary to current screening tests.

## Introduction

Loss of nociception (LON) at the feet is the necessary precondition (*conditio sine qua non*) for the diabetic foot syndrome (consisting of painless foot ulceration or Charcot osteoarthropathy) to develop. In diabetes mellitus, LON is due to axonal degeneration from distal to proximal zones, progressing insidiously over years of insufficient insulin action during the evolution of diabetic sensory neuropathy.^1^ Long rather than short A-delta and C nerve fibers are affected predominantly.

LON is greatly asymptomatic until a painless foot ulcer appears or the painless swelling of a foot from bone and joint damage (active Charcot foot). LON can be demonstrated by reduced numbers of nociceptors (intraepidermal nerve fiber endings) in skin biopsies,^2,3^ corresponding to increased perception thresholds of painful stimuli. Noninvasively, complete LON at the feet can be detected by demonstrating a high (>512 mN) pinprick-pain perception threshold^1,4^; whether a comparable “suprathreshold” heat-pain stimulus may serve the same purpose remains to be determined.

## Study Protocol, Materials, and Methods

### Study Design

This is a cross-sectional study of a 51°C heat-pain stimulus for diagnosing LON associated with diabetic foot ulceration (DFU), validated against a confirmed history of DFU (painless foot ulceration, reference standard), in comparison with 512-mN pinprick mechanical pain stimulus (diagnostic test). Both stimuli exceed the pain perception threshold in about 98% of healthy people^5^ and are, thus, considered equivalent “suprathreshold” pain stimuli for the great majority of the general population.

### Objective

To assess the performance of a 51°C heat-pain stimulation test for detecting severe DFU-related LON, simultaneously with 512-mN pinprick stimulation.

### Outcomes

Identification as diseased/not diseased according to the absence/presence of 51°C heat-pain perception; agreement with 512-mN pinprick-pain perception.

### Setting

A private outpatient diabetic foot clinic.

### Study Sample

A convenience sample of 72 German-speaking volunteers recruited from a group of patients and staff members, relatives, and friends. The sample size was chosen in accordance with previous research reports.^4,6^

## Materials and Methods

### Heat Stimulator

**Figure 1.**
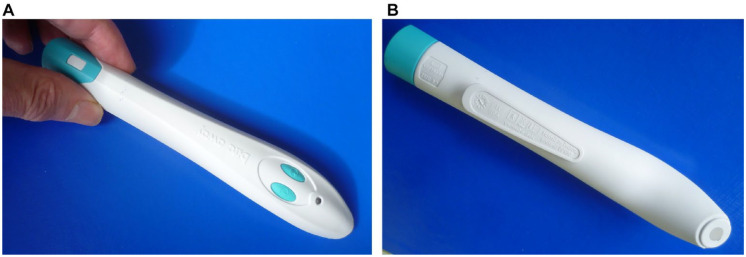
The bite away® device. (a) Upper side, showing activating buttons. (b) Lower side, showing heating plate (diameter 7 mm) on one end of the handle.

For delivering the 51°C heat-pain stimulus, a bite away® device (mibeTec G.m.b.H., 06796 Brehna, Germany; www.bite-away.com) was purchased in a local drug store. The bite away device has been marketed since 2006 for the local application of heat to treat insect bites and stings and is being sold all over the world. It is approved by the European Union as a medical device of class 2A (*noninvasive device intended for administration to the body, which exchanges energy with the patient in a therapeutic manner*).^7^ The device consists of a short plastic handle with a battery-driven electric heater inside and a circular 38.5-mm² ceramic heating plate at one end (see Figure 1a and 1b).

According to the instructions for use, the plate is pressed firmly onto the acute insect bite spot, and automatic heating of the plate is activated by a button on the upper side of the handle. Within about five seconds, the working temperature of 51°C (manufacturer’s specification) is reached, indicated by a light signal. (The working temperature was checked by infrared thermometry [QUIGG DSO 364, sensor diameter 3 mm; Shenzhen Dongdixin Technology Co., Ltd., Shenzhen, P. R. China].) According to a former supplier of bite away (Riemser AG, 17493 Greifswald, Germany) “its microchip-controlled time-heat-constant guarantees a maximum temperature of 51°C for either three or six seconds [ . . .], recognized by the patients as a very short and targeted induction of almost painful high temperature on the skin.”^7^ However, when bite away is applied to normal intact skin without insect bite, the heating evokes a short-lived, increasingly unpleasant and eventually truly painful burning or piercing sensation (distinguishable from flash-like, punctate pinprick pain),^6^ which subsides upon cessation of the heating (E.-A.C., own experience, unpublished). In fact, approximately 98% of healthy persons will perceive skin heating to 51°C as painful^5^ and as suprathreshold stimulation (which induces much higher pain ratings than threshold-level stimulation).^8^

Such heating for just six seconds is harmless but painful to normal adult skin; it will, however, damage the skin if applied for >15 minutes.^9^

#### Mode of action

Pain evoked by noxious high temperature (heat) is encoded and transduced mainly by C fiber, but also A-delta fiber, nociceptors.^10^ Due to the inborn individual equipment with nociceptors,^11^ age, and acquired individual psycho-physical irritability,^12^ pain perception thresholds vary considerably between persons, while there is relatively little intraindividual variability.^13^ Hence, in an unselected healthy population, heat pain perception thresholds range from 33°C to 53°C, with a bell-shaped normal distribution around an average of approximately 44°C.^5^

### Study Participants

There were three study groups.

Group I consisted of 30 control persons without diabetic neuropathy (healthy persons or persons with various health problems, including diabetes mellitus, see Table 1), eight of whom had already been assessed 42 months ago in an earlier study,^4^ with the 512-mN pinprick-pain stimulator.

**Table 1.** Clinical Characteristics of the Participants.

	Group I nonneuropathic controls	Group II neuropathic controls	Group III painless DFU
Study persons (study feet), n	30 (57 feet)	12 (24 feet)	30 (57 feet)
Gender, f:m, n	13:17	5:7	24:6
Age, years	74 (24-90)	75 (57-88)	71 (51-87)
Type 1:type 2 diabetes, n	3:12	0:12	2:28
Diabetes duration, years	14 (2-40)	18 (1-36)	22 (1-50)
Comorbidity			
Dialysis, n	1	0	1
Charcot foot, n	0	0	1
Minor amputation, n	1	0	5
Above-knee amputation, n	1	0	2
Radiculopathy, n	0	1	2
Unilateral plaster cast, n	1	0	0
Traumatic nerve damage, n	0	0	2
Peripheral artery disease, n	2	0	4
Painful neuropathy, n	0	2	2
Alcoholism, n	0	1	2
Occupational toxic exposure, n	0	0	1

Values are given as median (range).

Abbreviation: DFU, diabetic foot ulceration.

Group II consisted of 12 persons with established diabetic neuropathy according to accepted diagnostic criteria,^14^ such as Rydel-Seiffer tuning fork vibration amplitude sensation at the hallux <5/8 grade^15^ or absence of 10 g Semmes-Weinstein monofilament touch perception at the feet.^14^

Group III (reference standard) consisted of 30 persons with established diabetic neuropathy and a history of painless DFU.

Exclusion criteria were age <18 years, inability to comply with the experimental protocol, mental problems (eg, borderline personality disorder or drug addiction), comedication affecting sensory functions (eg, tranquilizers, painkillers), and skin pathology like keratosis palmoplantaris. The medical histories were taken from the patients’ files; the healthy controls were briefly interviewed. Clinical data of the participants are summarized in Table 1.

### Experimental Procedures

#### Preparations

The whole investigation took about five minutes to complete. Each study person was placed in supine position and bare-footed in a separate quiet room with the ambient temperature around 18°C. After accommodation, the person was familiarized by the examiner with the test procedure, the pain stimulators, and the numerical pain rating scale. The pinprick pain was compared with a toothpick pricking pain and the heat stimulus with the drinking temperature of a cup of coffee. The bite away device for treating insect bites or stings by concentrated heat had been used by, or was already known to, some of the study participants.

#### Explanation and ascertainment of stimulus responses

The sensations of interest “painful sting-like or burning discomfort” were explained in plain language. Every stimulation was accompanied by the examiner’s questions: “Does it hurt? And if so, how much?” Then, the pain intensity should be rated immediately on a numerical scale ranging from 0 to 10 (no pain at all, worst imaginable pain)^8^; this procedure was slightly different from that used in the earlier study.^4^

#### Pain rating

From a screen in front of them showing a 0 to 10 numerical scale with corresponding painless/painful facial expressions, participants were asked to choose a pain intensity from 0 to 10 and to tell it to the examiner for recording.

#### Procedure of stimulations

First, a 512-mN pinprick-pain stimulator made of fiberglass, as reported previously,^4^ was applied for one to two seconds, requiring careful handling by a skilled operator. Then, the heat-pain stimulus from the bite away device was applied until pain perception, for up to six seconds. Once switched on, the device stimulates automatically and is almost operator-independent.

Test areas near the second or third toe were stimulated, with a 10-second to 20-second interval: on the plantar side, the skinfold (pinprick stimulator) or the nearby foot sole (bite away) and, moreover, the dorsum of the foot adjacent to these toes (either stimulus). Thickened skin was avoided, as possible. Both feet were stimulated at random. The study persons were unaware of the sites of the foot to be stimulated. For control purposes, the palm of a hand was also stimulated. All stimulations were done by the same examiner (E.-A.C.). The study was carried out in accordance with the World Medical Association’s Declaration of Helsinki and the Ethics Commission of the Heinrich Heine University medical faculty (project no. 3718 “pain perception in diabetic neuropathy”). Written informed consent was provided by all study participants.

## Data Processing and Analysis

Data were analyzed per foot (unless stated otherwise), accounting for potential unilateral limb affections. The primary outcome was detection of DFU-associated LON by either stimulus, 51°C heat or 512-mN pinprick, and on either test area at the foot. The secondary outcome was the agreement between both stimulus responses. Descriptive statistics were performed. Sensitivity, specificity, positive and negative predictive values, Cohen’s κ, and Spearman’s ρ were calculated. The Mann-Whitney *U* test, χ^2^ test, and the McNemar test were applied, as appropriate. A two-tailed *P* < .05 was considered significant. Data are presented as median (range), unless indicated otherwise.

## Results

The experiments were well tolerated without any adverse effects. The demographic and clinical characteristics of the participants are summarized in Table 1. In group I and group III, three feet each were unavailable for examination due to amputation or plaster cast. Thus, in the 72 study participants, a total of 348 pairs (pinprick, heat) of pain scores were analyzed: 276 pairs of two foot test areas and 72 pairs of a hand test area. Numerical pain rating was deemed reliable, according to the performance of eight healthy control persons, whose hands and feet had been stimulated with a 512-mN pinprick stimulus 42 months before. Their current and previous pain perception scores were correlated (*r* =0.45) and were not significantly different (*P* > .05; *U* test), which is in keeping with earlier observations.^4^

### Pain Perception Scores

#### Nonneuropathic control group (group I, n = 57 legs)

In this group, 51°C heat stimulation evoked greater pain than 512-mN pinprick stimulation, pain scores differed significantly. Details are summarized in Table 2. The association between heat and pinprick pain scores was significant at hand (*r* = 0.45) and feet (*r* = 0.51 plantar and *r* = 0.53 dorsal). The agreement between heat and pinprick pain score of 0 (vs > 0) was “moderate” to “perfect” (Cohen’s κ, 0.46-1) at hand and feet, respectively.

**Table 2.** Stimulus Responses (Pain Intensity Rated 0-10) at Different Test Areas.

Study group, number of feet	Stimulus	Pain	Ratings 0 to 10	Foot dorsal side^a^
		Hand^b^	Foot plantar side^a^	
(I) Controls, PNP-negative, 57 feet	51°C heat	6.5 (0-10)	7 (0-10)	8.5 (2-10)
	512 mN pinprick	3.5 (0-9.5)	4 (0-9)	3 (0-10)
(II) Controls, PNP-positive, 24 feet	51°C heat	5.5 (0-8)	4.5 (0-8.5)^c^	4 (0-10)^c^
	512 mN pinprick	2 (0-6)	2 (0-6)^c^	2 (0-7)^c^
(III) DFU, PNP-positive, 57 feet	51°C heat	6 (0-10)	0 (0-8)^d^	0 (0-10)^d^
	512 mN pinprick	3 (0-10)	0 (0-4)^d^	0 (0-6)^d^

Values are given as median (range). Significant differences (*P* < .05; *U* test).

Abbreviations: DFU, diabetic foot ulceration; PNP, polyneuropathy.

a In group I, foot pain scores between heat and pinprick.

b In all groups, hand pain scores between heat and pinprick.

c Heat and pinprick foot pain scores versus groups I and III.

d Heat and pinprick foot pain scores versus groups I and II.

#### Neuropathic control group (group II, neuropathy DFU-negative, n = 24 legs)

The heat stimulation evoked greater pain than pinprick at the hand test area only (*P* < .05), whereas the difference at the foot test areas was not significant, see Table 2. Stimulated pain at the feet was rated significantly lower than that in group I (Table 2). The association between heat and pinprick pain scores was significant only at the feet (*r* = 0.52 plantar and *r* = 0.79 dorsal). The agreement between heat and pinprick pain score of 0 (versus > 0) was “fair” (Cohen’s κ, 0.29-0.34).

#### Standard reference group (group III, neuropathy DFU-positive, n = 57 legs)

Stimulated pain at the feet was rated significantly lower than that in groups I and II, see Table 2. The association between heat and pinprick pain scores was significant only at the feet (*r* = 0.4 plantar and *r* = 0.55 dorsal). The heat stimulation evoked greater pain than pinprick only at the hand test area (*P* < .05), whereas the difference at the foot test areas was not significant, see Table 2. The agreement between heat and pinprick pain score of 0 (versus > 0) was “fair” to “substantial” (Cohen’s κ, 0.35-0.63).

### Detection of DFU-Associated LON

A heat-pain perception threshold > 51°C (revealed by the absence of pain perception, equal to a pain score of 0, in response to 51°C heat stimulation, test positive) at the dorsal side of the foot was found in 37 of the 57 feet with DFU-related LON (20/57 = 35% false negatives), while a pain score > 0 was found in 57 of the 57 feet without any neuropathy (no false positives). At the plantar side, a pain score of 0 (positive test) was found in 46 of the 57 feet with DFU-related LON (11/57 = 19% false negatives), and a score >0 in 53 of the 57 feet without neuropathy (4/57 = 7% false positives) (*P* < .0001). The positive likelihood ratio was 11.5.

A pinprick-pain perception threshold >512 mN (a pain score of 0 in response to 512-mN pinprick stimulation, positive test) at the dorsal side of the foot was found in 44 of the 57 feet with DFU-related LON (13/57 = 23% false negatives), and a pain score >0 was found in 54 of the 57 feet without any neuropathy (3/57 = 5% false positives). At the plantar side, a pain score of 0 was found in 51 of the 57 feet with DFU-related LON (6/57 = 10% false negatives), and a score >0 in 52 of the 57 feet without neuropathy (5/57 = 9% false positives) (*P* < .0001). The positive likelihood ratios were 14.5 and 10.2, respectively.

Proportions of pain score 0 were significantly different between heat and pinprick stimulation only for the dorsal side of the foot (McNemar’s test *P* < .05), but not for the plantar side. Proportions of pain score 0 were significantly different between group III, and groups I and II, respectively. For further details, see Tables 3 and 4.

**Table 3.** Stimulus Response 0 on Numerical Pain Rating Scale 0 to 10, Proportions of Feet Scored 0.

Study group, number of feet	Stimulus	Plantar side	Dorsal side
(I) Controls, PNP-negative, 57 feet	51°C heat	4/57	0/57
	512 mN pinprick	5/57	3/57
(II) Controls, PNP-positive, 24 feet	51°C heat	9/24	6/24
	512 mN pinprick	7/24	7/24
(III) DFU, PNP-positive, 57 feet	51°C heat	46/57*	37/57*
	512 mN pinprick	51/57*	44/57*


Abbreviations: DFU, diabetic foot ulcer; PNP, polyneuropathy.

* *P* < .0001 versus non-DFU feet, χ^2^ test.

**Table 4.** Test Quality of Pain Perception Thresholds >51°C (Heat) or >512 mN (Pinprick) to Detect DFU-Related LON.

Pain perception threshold	Sensitivity	Specificity	PPV/NPV^a^	Accuracy^a^
Plantar				
>51°C heat	80.70%	92.98%	56.10%/97.75%	91.75%
>512 mN pinprick	89.47%	91.23%	53.12%/98.73%	91.05%
Dorsal				
>51°C heat	64.91%	100.00%	100.00%/96.25%	96.49%
>512 mN pinprick	77.19%	94.74%	61.97%/97.39%	92.98%

Abbreviations: DFU, diabetic foot ulceration; LON, loss of nociception; NPV, negative predictive value; PPV, positive predictive value.

a Based on disease (DFU-related LON) prevalence of 10% in the German diabetic population (authors’ estimate).

## Discussion

The study shows that both stimuli, 51°C heat pain and 512-mN pinprick pain, were effective in detecting DFU-related LON; hence, the data corroborate earlier test data obtained with 512-mN pinprick stimulus alone.^4^ In the present study, however, sensitivity and specificity of both tests were less favorable than those that could have been expected from the earlier study. We attribute this discrepancy to the different pain-perception-ascertainment methods used in the present study. While, in the earlier study, the robust yes/no method with only two possible results, normal or abnormal, was used, in the current study, the stimulus responses had to be chosen from a 0 to 10 continuum of possible pain perception intensities (which may have caused a bias toward estimating > 0 grade). Moreover, looking on the rating scale panel (instead of having the eyes blindfolded) may have distracted the attention of the probands from deciding whether it was pain or no pain what they were feeling. On the other hand, the cases with DFU-related LON and pain score > 0 suggest that any relative increase in individual heat pain perception threshold below 51°C might predispose for a painless DFU, rather than the absolute increase to above 51°C (and, hence, relative rather than absolute LON).

To clarify these points, a reassessment using a proper stimulus-response ascertainment is highly desirable.

In the nonneuropathic limbs, a 51°C heat stimulus evoked a significantly greater pain intensity than a 512-mN pinprick stimulus, and the heat feeling was distinctly painful. This phenomenon may be due to the device’s continuously rising temperature (until reaching 51°C), which could be noticed before reaching the individual pain threshold: a gradually increasing and pleasant warm sensation (in healthy persons, warm detection threshold is about 5°C-8°C below the heat pain threshold on average),^2,16^ which changed abruptly into an unpleasant heat-pain sensation. (Some nonneuropathic participants reported feeling an “after-glow,” fading in about 10 seconds, as was described by Lele in 1954.)^17^

By contrast, pinprick stimulation, a single punctate stimulation of one-second to two-second duration, evoked a sudden and short pricking pain sensation, which sometimes was difficult to discern from mere pressure discomfort (participants’ spontaneous comments, unpublished).

This difference in pain quality and quantity might also be related to the greater contact area of the heat-pain stimulus than that of the pinprick stimulus (38.5 mm² vs 0.091 mm²) and, hence, to the effect of spatial summation.^8,18^ Of note, DFU-related LON abolished this difference in stimulus response between both stimuli.

The 512-mN pinprick-pain stimulator had been assessed previously, with optimal results.^4^ In the present study, however, its performance was less convincing: 89% sensitivity and 91% specificity at the plantar side (vs 99.5% sensitivity and 99.4% specificity, as reported previously).^4^ This difference is likely due to the various methods of stimulus response ascertainment, as outlined above. Furthermore, the poor performance at the dorsal side of the foot (sensitivity 77%, specificity 94%) may be related to the particular nerve supply of that test area.

What is the diagnostic ability of the heat-pain test, as it stands now? How good is it at predicting DFU-related LON? The high specificity of 92% to 100% (and positive likelihood ratios > 10) is particularly interesting as it is considerably higher than that reported in the literature for other sensory tests to detect DFU-related neuropathy: vibration amplitude perception threshold by biothesiometer ≥25 V (86% sensitivity, 56% specificity)^19^ or by Rydel-Seiffer tuning fork <5/8 (97% sensitivity, 62% specificity)^20^; touch perception of 10 g by Semmes-Weinstein monofilament (91% sensitivity, 34% specificity).^19^ High specificity is desirable when there is a need to avoid false positives.^21^ For instance, a positive heat-pain test in a patient with an elevated vibration perception threshold (<5/8 Rydel-Seiffer tuning fork) increases the specificity from 62% (tuning fork) to 97% to 99.9% (combined tests), which is high enough to make the diagnosis of DFU-related LON without further assessments.^22^ In other words, in a patient with a probability of DFU-associated LON of 50% (roughly the prevalence in the German population with diabetic neuropathy according to tuning fork <5/8),^23^ a positive bite away test (pain score 0) will update the probability of having the condition to 90%, by Fagan’s nomogram.^24^ The high specificity of the heat-pain test corresponds to a relatively low sensitivity of 64% to 80%; hence, if considered for screening purposes, a high rate of 17% to 26% of false negative tests has to be anticipated.

### Limitations of the Study

Although the findings are consistent with basic research data,^8,9,17,18,25^ some weaknesses of the study design have to be considered as potential confounders. First, there was a problem with the pain ascertainment, as discussed above, and second, the assessments were not independent as the clinical diagnoses of the participants were known to the examiner. Furthermore, in cases with peripheral artery disease and very cold feet, the device’s heating time could probably have been too short to raise skin temperature to the level of 51°C. Moreover, a low nerve temperature attenuates signal transmission and raises perception thresholds (nerve warming has opposite effects).

## Conclusions and Clinical Implications

In the present study, which is considered preliminary, the bite away 51°C heat-pain test detected DFU-related LON with high specificity and relatively low sensitivity. Hence, it may complement conventional diagnostic procedures suffering from low specificity. Further studies using a proper stimulus response ascertainment are required to fully explore its potential as a diagnostic test.
